# Data on Arsenic(III) removal using zeolite-reduced graphene oxide composite

**DOI:** 10.1016/j.dib.2019.01.004

**Published:** 2019-01-10

**Authors:** Richa Soni, Dericks Praise Shukla

**Affiliations:** School of Engineering, Indian Institute of Technology, Mandi 175005, Himachal Pradesh, India

**Keywords:** Adsorption, Arsenic, Isotherm, Kinetics

## Abstract

This data article proposes a mechanisms of Arsenic(III) removal from water using zeolite-reduced graphene oxide (ZrGO) composite. Here, the adsorption kinetic and adsorption isotherm analysis for the data obtained by removal of Arsenic(III) using ZrGO is presented. The kinetic model fits the pseudo second order kinetics and indicates the adsorption mechanism to be chemisorption. Redlich Peterson isotherm model best describes the adsorption isotherm. The data are related to the research article “Synthesis of Fly ash based zeolite-reduced graphene oxide composite and its evaluation as an adsorbent for arsenic removal” (Soni and Shukla, 2019).

**Specifications table**

**Table:** 

Subject area	Environmental engineering
More specific subject area	Adsorption
Type of data	Figure, table
How data were acquired	The uptake of Arsenic(III) by the adsorbent (q_e_) was determined based on the subtraction of the initial and final concentration of adsorbate. Arsenic concentration was measured using both portable Metalyser HM-1000 and ICP-MS (Thermo Scientific X series2)
Data format	Analyzed
Experimental factors	ZrGO was added to the contaminated water in a beaker and the solution was stirred for varying time interval. After the treatment process, the samples were collected at set time intervals from the beaker, filtered using 0.45 µm nylon membrane filter and analyzed for residual arsenic solution.
Experimental features	ZrGO was used as an adsorbent for removal of Arsenic(III) from water. Varying concentrations of Arsenic(III) solutions were prepared in the laboratory by diluting arsenite stock solution.
Data source location	Mandi, Himachal Pradesh, India
Data accessibility	Data are included in this article
Related research article	R. Soni, D. P. Shukla, Synthesis of Fly ash based zeolite-reduced graphene oxide composite and its evaluation as an adsorbent for arsenic removal, Chemosphere. [1]

**Value of the data**

• Adsorption isotherms give an insight into the interaction of Arsenic(III) with ZrGO which explains the removal process.
• The isotherm and kinetic analysis will be useful for predicting the adsorption capacity, modeling and mechanism of Arsenic(III) using ZrGO.
• These data can be important for removal of Arsenic(III) from aqueous solution.

## 1 Data

**Fig. 1:**
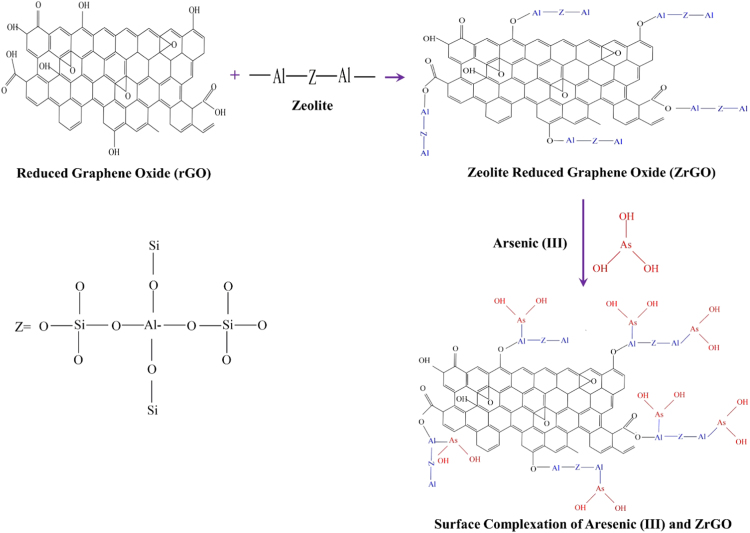
Proposed schematic illustration of the surface complexation reaction of ZrGO composite and Arsenic(III).

Fig. 1 illustrates a possible schematic of the reaction of ZrGO composite and Arsenic(III) which depicts the removal of arsenic by surface complexation at the aluminium present in the zeolites. Authors would like to add that removal of arsenic using ZrGO composite is a complex process and involves a lot of mechanisms and hence explaining all in a single chemical equation is not possible. Therefore, authors have tried to propose a basic hypothesis of the removal as shown in Fig. 1.

The arsenic adsorption on zeolites is the result of exchange between terminal aluminol or silanol hydroxyl groups and adsorbate anionic species. The mechanism of Arsenic(III) adsorption on rGO based composites should be interpreted as surface complexation modeling [2].

## 2 Experimental design, materials, and methods

Adsorption of Arsenic(III) from synthetic aqueous solution using ZrGO was performed in batch experiments. Arsenic(III) stock solution (1000 µg/L) was prepared by dissolving arsenic trioxide in hydrochloric acid and DI. Arsenic working solutions were freshly made by diluting the stock arsenic solution until desired concentration using DI. Here, experiments were performed on three varying concentrations of Arsenic(III); 100, 200 and 300 µg/L. The adsorbent (ZrGO) was added to 50 mL of working arsenic solution and stirred at 600 rpm for a fixed time. After the treatment, the samples were collected from the beaker, filtered using 0.45 µm nylon membrane filter and analyzed for residual arsenic solution.

### 2.1 Adsorption kinetics

The amount of arsenic adsorbed by ZrGO was determined using a mass balance equation expressed in Eq. (1) [3].

(1) $$q_{e}=V\frac{(C_{o}-C_{e})}{W}$$

where q_e_ is arsenic concentration adsorbed on the ZrGO at equilibrium (µg/g), C_o_ is arsenic concentration in solution (µg/L), C_e_ is arsenic concentration in solution after treatment (µg/L), V is volume of solution used (L) and m is mass of ZrGO used (g).

The dynamics of the adsorption process can best be understood by the processing of kinetics adsorption data. Moreover, it helps in prediction of adsorption rate, which gives information for designing and modelling of the process [4]. Also, adsorption kinetics is of great significance to evaluate the performance of a given adsorbent and gain insight into the underlying mechanisms. Here, the data are fit into pseudo first order, pseudo second order and intraparticle diffusion kinetic model and their equations are presented in Eqs. (2), (3), (4) respectively.

(2) $$\log(q_{e}-q)=\log{(q}_{e})-\frac{k_{1}t}{2.303}$$

(3) $$\frac{t}{q}=\frac{1}{k_{2}q_{e}^{2}}+\frac{t}{q_{e}}$$

(4) $$q_{t}=k_{\mathrm{dif}}t^{0.5}+C$$

where q is the amount of arsenic adsorbed on the adsorbent at time t (min), k_1_ (min^−1^) is the rate constant of first-order adsorption, k_2_ is the rate constant of the second-order adsorption, C is the intercept and K_dif_ (µg/g/min^−0.5^) is the intraparticle diffusion rate constant. If the first order kinetics is applicable, a plot of log (q_e_-q) vs t will provide a linear relationship; if the second order kinetics is applicable, a plot of t/q_t_ vs t will be a linear relationship, if the and for intraparticle diffusion the plot of q_t_ v/s t^0.5^ will hold a linear relationship.

**Fig. 2:**
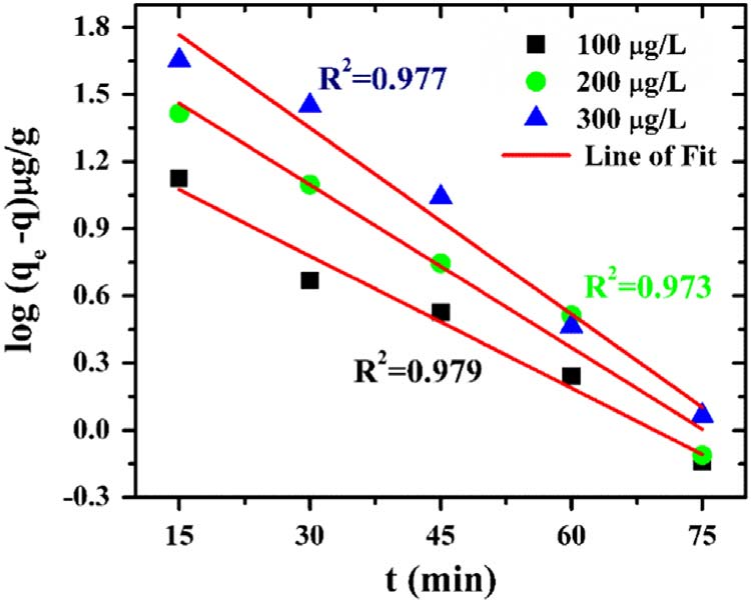
Pseudo first order model for arsenic adsorption on ZrGO.

**Fig. 3:**
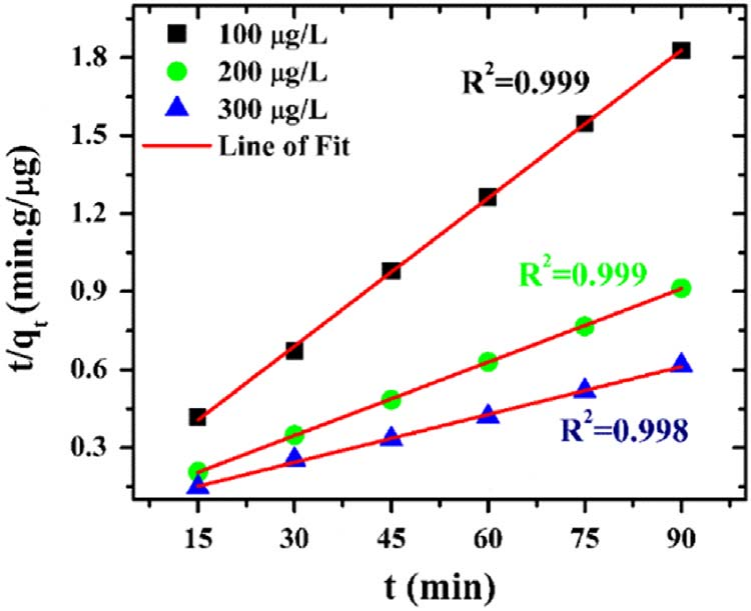
Pseudo second order model for arsenic adsorption on ZrGO.

**Fig. 4:**
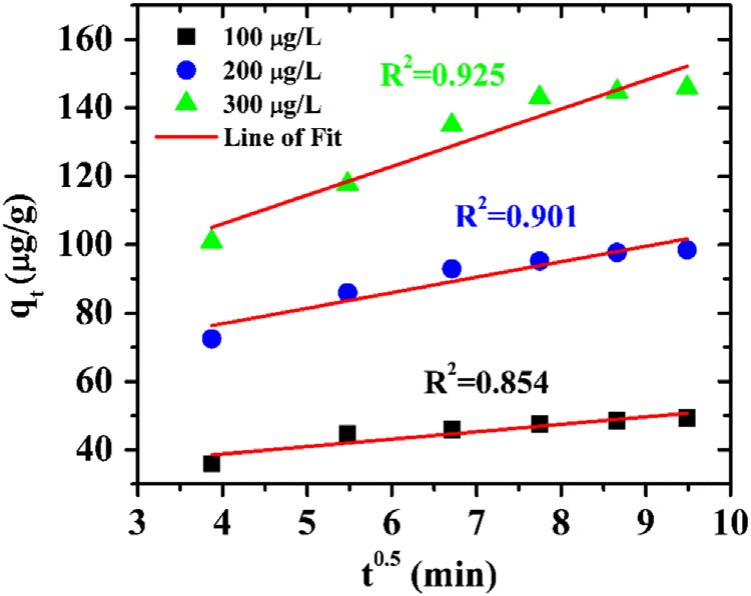
Intraparticle diffusion model for arsenic adsorption on ZrGO.

For this data article, the plots were made for three initial arsenic concentrations (100, 200, 300 µg/L). The experimental data were fitted in the mentioned three kinetic models. The average R^2^ value for pseudo first order kinetic model is 0.976, for pseudo second order kinetic model it is 0.999 and for intra-particle diffusion it is 0.894. The results show that pseudo second order kinetic model gave the best fit and the graph is presented. The results are presented in Fig. 2, Fig. 3, Fig. 4. These results indicate that the adsorption system belongs to the pseudo second-order kinetic model. Similar results have been obtained by Khatamian et al. [5]. In this model, the rate-limiting step is the surface adsorption that involves chemisorption, where the removal from a solution is due to physicochemical interactions between the two phases [6]. Liu et al. [7] has also mentioned that pseudo-second order kinetic assumes that chemisorption controls the adsorption rate.

### 2.2 Adsorption isotherms

The experimental data were fitted with Langmuir, Freundlich and Redlich–Peterson isotherms. The experimental q_e_ was calculated by Eq. (1) and the predicted q_e_ is obtained from the non-linear expressions of Langmuir [8] Freundlich [9] and Redlich–Peterson [10] isotherms as shown in Table 1. In the case of the non-linear method, a trial-and-error procedure was developed to determine the isotherm parameters using an optimization routine to maximize the coefficient of determination between the experimental data and isotherms [11]. This was performed in the solver add-in with Microsoft׳s spreadsheet, Microsoft Excel. The protocol which has been used is, experimental data were manually entered in MS-Excel, the formulated algorithm was carried out and the predicted curve was overlaid on the experimental data points, and goodness of fit was observed [12], [13].

**Table 1:** Isotherm parameters.

***Isotherms***	***Langmuir***	***Freundlich***	***Redlich Peterson***
**Non-linear expression**	$q_{e}=\frac{q_{\max}K_{l}C_{e}}{1+K_{l}C_{e}}$	$q_{e}=K_{f}C_{e}^{1/n}$	$q_{e}=\frac{{\mathrm{AC}}_{e}}{1+{\mathrm{BC}}_{e}^{g}}$
**RMSE**	8.801	28.888	3.674
**x**^**2**^	0.947	10.199	0.165
**R**^**2**^	0.921	0.150	0.986

**Fig. 5:**
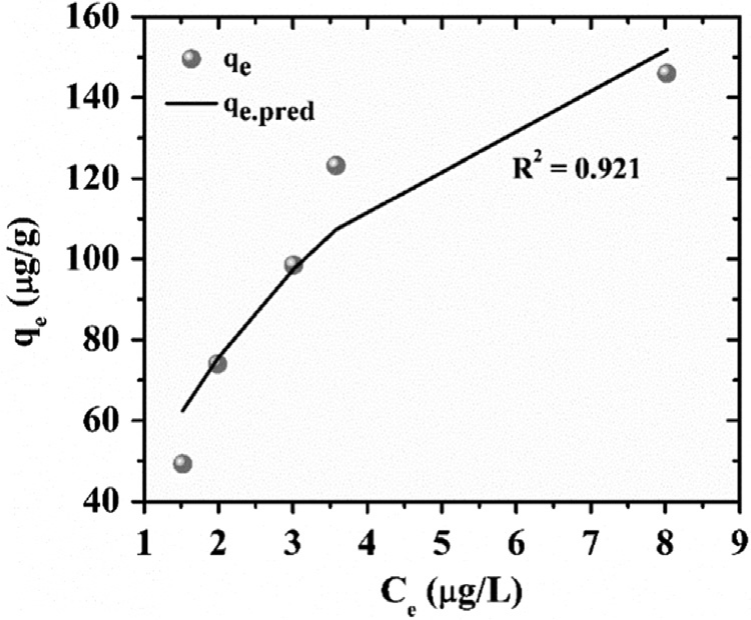
Langmuir isotherm fitting for residual arsenic after ZrGO dosing.

**Fig. 6:**
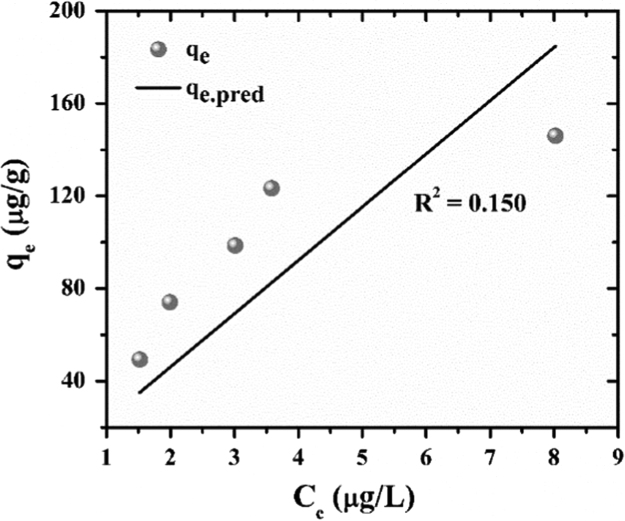
Freundlich isotherm fitting for residual arsenic after ZrGO dosing.

**Fig. 7:**
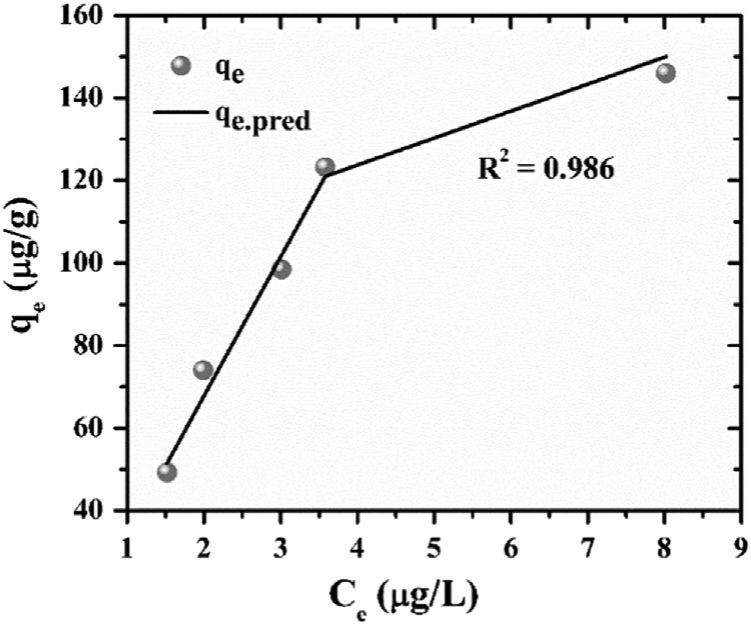
Redlich Peterson isotherm fitting for residual arsenic after ZrGO dosing.

The results for goodness of fit for the three isotherms have been presented in Table 1. The adsorption isotherm fitting results are presented in Fig. 5, Fig. 6, Fig. 7. The R^2^ values of Redlich–Peterson is the best, 0.986 (Fig. 7). Redlich–Peterson isotherm has features of both Langmuir and Freundlich [9]. Langmuir isotherm indicates monolayer adsorption and homogeneous adsorption, with no transmigration of the adsorbate in the plane to the surface [9]. Freundlich isotherm indicates non-ideal and reversible adsorption, not restricted to the formation of monolayer [9]. The Redlich-Peterson model has a linear dependence on concentration in the numerator and an exponential function in the denominator [14] to represent adsorption equilibria over a wide concentration range, which can be applied either in homogeneous or heterogeneous systems due to its versatility [15]. Thus, it can be concluded that adsorption of arsenic to ZrGO is hybrid mechanism and does not follow ideal monolayer adsorption.
